# Allergens in red ginseng extract induce the release of mediators associated with anaphylactoid reactions

**DOI:** 10.1186/s12967-017-1249-x

**Published:** 2017-06-28

**Authors:** Lu Wang, Yazheng Zhao, Ye Yang, Yuanjia Hu, Xiaohan Zou, Boyang Yu, Jin Qi

**Affiliations:** 1 State Key Laboratory of Natural Medicines, China Pharmaceutical University, Nanjing, China; 2 Jiangsu Key Laboratory of TCM Evaluation and Translational Research, China Pharmaceutical University, Nanjing, China; 3 State Key Laboratory of Quality Research in Chinese Medicine, Institute of Chinese Medical Sciences, University of Macau, Macao, China

**Keywords:** Red ginseng, Anaphylactoid reaction, Mast cell degranulation, RBL-2H3 cell extraction

## Abstract

**Background:**

Anaphylactoid reactions induced by preparations containing red ginseng have been reported. The aim of this study is to evaluate the allergenicity and screen potential allergens in red ginseng extract thoroughly.

**Methods:**

Red ginseng extract (RGE) and different fractions of RGE were prepared and evaluated by measuring the degranulation and viability of rat basophilic leukemia 2H3 (RBL-2H3) cells. Potential allergens were screened by RBL-2H3 cell extraction and allergenicity verified in RBL-2H3 cells, mouse peritoneal mast cells, Laboratory of Allergic Disease 2 (LAD2) human mast cells and mice, respectively.

**Results:**

80% ethanol extract of red ginseng extract induced mast cell degranulation with less cytotoxicity, but 40% ethanol extract could not. Ginsenoside Rd and 20(*S*)-Rg3 could induce a significant increase in β-hexosaminidase release, histamine release and translocation of phosphatidylserine in RBL-2H3 cells. Ginsenoside Rd and 20(*S*)-Rg3 also increased β-hexosaminidase release and the intracellular Ca^2+^ concentration in mouse peritoneal mast cells and LAD2 cells. In addition, histamine levels in serum of mice were elevated dose-dependently.

**Conclusions:**

Ginsenoside Rd and 20(*S*)-Rg3 are potential allergens that induce the release of mediators associated with anaphylactoid reactions. Our study could guide optimization of methods associated with Rd/20(*S*)-Rg3-containing preparations and establishment of quality standards for safe application of Traditional Chinese Medicines.

## Background

Ginseng (*Panax ginseng* C.A. Meyer) has been used as an elixir in Asia for thousands of years. Additionally, it is one of the most popular herbal medicines used as a functional food or therapeutic supplement in Western countries [1, 2]. Red ginseng is a very popular processed product of ginseng with different biologic activities and wide clinical applications. During the steaming or heating, less polar ginsenosides (20(*S*)-Rg3, Rh2, and Rs3, etc.) in red ginseng increased [3], which have been reported to exhibit greater potential than polar ginsenosides in terms of anti-inflammatory, anti-oxidative and anti-cancer effects [4, 5]. Therefore, an extract of red ginseng or its constituents are used more often in some TCM formulations than those of other processed products for the treatment of cardiovascular diseases or cancer [6, 7]. This is especially the case for Traditional Chinese Medicine Injections (TCMIs) [8] such as shengmai injection and shenmai injection. However, along with the wide use of red ginseng, adverse drug reactions occur frequently, especially anaphylactoid reactions (ARs). Several red ginseng-containing TCMIs [9] or ginsenoside-containing TCMIs [10] have been shown to cause ARs that may be related to components in red ginseng. Moreover, several cases of ARs induced by oral administration of ginseng or inhalation of ginseng dust have also been recently reported [11, 12]. Therefore, the allergenicity and potential allergens in red ginseng (especially its bioactive components) must be clarified to improve preparation methods and quality as well as to ensure safety of use.

Due to the complexity of components in TCMs, the screening and analysis of specific bioactive components in TCMs are troublesome issues. Pharmacologic studies have shown that most drugs played a role firstly via combination with some receptors or channels on cell membranes. Therefore, cell membrane chromatography and cell membrane extraction have been applied in many studies and have some advantages over conventional methods (which are time-consuming and arduous) for preliminary investigation of the potential bioactive components of TCMs [13].

In this report, the allergenicity of red ginseng extract (RGE) and different extract fractions were preliminarily evaluated in rat basophilic leukemia 2H3 (RBL-2H3) cells. Potential allergens in RGE were screened using RBL-2H3 cell extraction and the allergenicity of target components was verified further in mouse peritoneal mast cells, Laboratory of Allergic Disease 2 (LAD2) human mast cells in vitro and mice in vivo.

## Methods

### Reagents and materials

Modified Eagle’s medium (MEM), StemPro-34 SFM Medium and trypsin were purchased from Gibco (Grand Island, NY, USA). Fetal bovine serum (FBS) was obtained from Sciencell (Carlsbad, CA, USA). Recombinant human stem cell factor was purchased from Peprotech (Rocky Hill, NJ, USA). Compound 48/80 (C48/80), l-glutamine, 3-(4,5-Dimethylthiazol-2-yl)-2,5-diphe-nylte-trazolium bromide (MTT), *p*-nitrophenyl-N-acetyl-β-d-glucosaminide and poly-d-lysine hydrobromide (PDL) were purchased from Sigma Aldrich (Saint Louis, MO, USA). Triton X-100 was obtained from Amresco (Solon, OH, USA). Penicillin and streptomycin were purchased from Beyotime Biotechnology (Shanghai, China). Ginsenoside Rd, Rg1, Rf, 20(*S*)-Rg3 and 20(*R*)-Rg3 were purchased from Must Bio-technology (Chengdu, China). Pluronic acid F-127 was obtained from Invitrogen (Carlsbad, CA, USA). A calcium 5 assay kit was obtained from molecular devices (Sunnyvale, CA, USA). ELISA kits for histamine were purchased from Biocalvin (Suzhou, China). An Annexin V-FITC fluos staining kit was purchased from KeyGEN BioTECH (Nanjing, China).

### Cells

RBL-2H3 MCs were purchased from the cell bank of the Chinese Academy of Sciences (Shanghai, China). Cells were cultured in MEM supplemented with 15% FBS, 100 U/ml penicillin, 100 μg/ml streptomycin and 0.11 g/l sodium pyruvate in a humidified atmosphere of 5% CO_2_ at 37 °C.

Laboratory of Allergic Disease 2 (LAD2) human MCs were provided by Dr. Renshan Sun at the Third Military Medical University (Chongqing, China). Cells were cultured in StemPro-34 SFM medium supplemented with 2 mM l-glutamine, 100 U/ml penicillin, 100 μg/ml streptomycin and recombinant human stem cell factor (100 ng/ml). Cell suspensions were seeded at 1.0 × 10^5^ cells/ml and maintained in a humidified atmosphere of 5% CO_2_ at 37 °C. Cell culture medium was hemi-depleted every week with fresh medium.

### Animals

ICR male mice (18–22 g) were obtained from Yangzhou University (Yangzhou, China). Animals were housed with a 12-h light–dark cycle at 22 °C and relative humidity of 55 ± 5%, and had free access to food and water. All experiments protocols involving animals were carried out according to guidelines set by Institutional Animal Care and Use Committee of China Pharmaceutical University (Jiangsu, China).

### Isolation of mouse peritoneal mast cells (MPMC) for validation of potential allergens

MPMCs were isolated using a method adapted from Jensen et al. [14]. Adult male mice were sacrificed. A total of 10 ml ice-cold phosphate-buffered saline (PBS) was used to make two sequential peritoneal lavages, which were combined and centrifuged at 800*g* for 10 min at 4 °C. The supernatant was decanted to leave a small MC pellet (≈2 ml), and 2 ml of 30% Percoll and 80% Percoll added successively to form an interface (final volume ratio: 1:1:1). After centrifugation at 600*g* for 15 min at 4 °C, cells at the junction interface were collected and washed with PBS twice (200*g*, 10 min, 4 °C). MCs were resuspended in DMEM with 10% FBS. Purity and viability were determined by toluidine blue staining and trypan blue exclusion.

### Preparation of RGE and RGE fractions

Red ginseng was soaked in 95% ethanol overnight, and then extracted thrice under reflux for 3, 3 and 2 h, respectively. The filtrate was evaporated under reduced pressure at 50 °C, and the residue was suspended in H_2_O and freeze-dried to obtain RGE.

RGE was dissolved in water and absorbed by AB-8 macroporous resin for 1 h. After elution with water [five times the column volume (5 V)] and 25% ethanol (4 V), the resin was eluted with 40% ethanol (3 V). The eluate was collected, concentrated, and freeze-dried to obtain 40% ethanol extract (40% EE). After elution a further four times with 40% ethanol, the resin was eluted with 80% ethanol (5 V). Then the eluate was collected, concentrated and freeze-dried to obtain 80% ethanol extract (80% EE).

### RBL-2H3 cell extraction

RGE was dissolved in MEM without FBS and filtered through a membrane (pore size, 0.22 μm) to create the sample solution. After incubation of RBL-2H3 cells with the filtrate at 37 °C and 5% CO_2_ for 1 h, the supernatant was discarded. Deposited cells were washed six times with 3 ml of PBS each time. Eluates were discarded except the last one, which was collected and used as a control for HPLC–DAD-Q-TOF–MS/MS analyses. Then, deposited cells were denatured and extracted with 3 ml of 80% methanol. After centrifugation at 9000*g* for 10 min, the supernatant was collected and dried. The residue was dissolved in methanol again and filtered through a membrane (pore size, 0.45 μm) for HPLC–DAD-Q-TOF–MS/MS analyses [15]. Cells incubated with MEM without FBS were prepared to the control sample using the same procedures as described above.

### HPLC–DAD-Q-TOF–MS/MS analysis for identification of components combined with RBL-2H3 cells

HPLC–DAD-Q-TOF–MS/MS analyses were carried out using 1260 Infinity HPLC instrument (Agilent Technologies, Santa Clara, CA, USA) coupled with a 6520 Q-TOF mass spectrometer (Agilent Technologies) equipped with a dual electrospray ionization (ESI) source. The mobile phase consisted of water-0.01% formic acid (A) and acetonitrile-0.01% formic acid (B). Gradient elution conditions were: 0–30 min, 8–25% B; 30–80 min, 25–45% B; 80–100 min, 45–76% B and then returned to the initial condition. The flow rate was 1 ml/min. Chromatographic separation was carried out at 30 °C on a Venusil C_18_ column (250 mm × 4.6 mm, 5 μm). Samples were detected at 203 nm. Parameters of MS detection were optimized as follows: drying gas temperature, 325 °C; flow rate of drying gas (N_2_), 8.0 l/min; fragmentor voltage, 120 V; nebulizer, 40 psi; capillary, 3500 V; skimmer, 65 V; Oct RFV, 750 V. The sample collision energy was set at 35 V. All acquisition and analyses of data were controlled by Mass Hunter software (Agilent Technologies). Mass spectra were recorded in the range *m/z* 100–1700 with accurate mass measurement of all peaks. Each sample was analyzed in negative mode.

### Measurement of cell viability

In order to assess that β-hexosaminidase release was induced due to allergenicity of potential allergens rather than cytotoxicity. Cell viability was determined by the MTT assay. RBL-2H3 cells were seeded in a 96-well plate at 1 × 10^4^ cells/well. After 24 h of incubation at 37 °C in an atmosphere of 5% CO_2_, cells were treated with different concentrations of test compounds for 30 min. Then, the supernatant was discarded and cells were incubated with MEM containing 0.5 mg/ml MTT at 37 °C and 5% CO_2_. After 3 h, the medium was removed and 150 μl dimethyl sulfoxide added to each well. Absorbance was measured at 570 and 650 nm.

### Measurement of β-hexosaminidase release

β-Hexosaminidase is an enzyme contained in the cytoplasmic granules of mast cells and the level of this enzyme released into the supernatant provides an indication of the degree of degranulation when mast cell is activated. β-Hexosaminidase release from mast cells was examined as described previously with some modifications [9].

RBL-2H3 cells (1 × 10^4^ cells/well) or MPMCs (3 × 10^4^ cells/well) in a 96-well plate were cultured for 24 h, washed with MEM/DMEM without FBS, respectively, and stimulated with different concentrations of sample solutions or the positive control C48/80 (30 μg/ml) for 30 min at 37 °C in an atmosphere of 5% CO_2_.

LAD2 cells were resuspended at 3 × 10^5^ cells/ml in HEPES buffer (10 mM HEPES, 137 mM NaCl, 2.7 mM KCl, 0.38 mM Na_2_HPO_4_·7H_2_O, 5.6 mM glucose, 1.8 mM CaCl_2_·H_2_O, 1.3 mM MgSO_4_·7H_2_O, pH 7.4). Cell suspension (150 μl) was added to each well of 96 wells, and then stimulated with different concentrations of sample solutions or the positive control C48/80 (1.5 μg/ml) for 30 min at 37 °C in an atmosphere of 5% CO_2_ [16].

β-Hexosaminidase released into supernatants and cell lysates was quantified by hydrolysis of *p*-nitrophenyl-N-acetyl-β-d-glucosamide in 0.1 M sodium citrate buffer (pH 4.5) for 60 min at 37 °C. Absorbance of each well was measured at 405 nm. Percentage of release of β-hexosaminidase was calculated as a percentage of total content, using the following formula:

$$\beta{\text{-hexosaminidase}}\left( {\text{\%}} \right) = \frac{{{\text{OD}}\left( {\text{supernatant}} \right)}}{{{\text{OD }}\left( {\text{supernatant}} \right) + {\text{OD }}\left( {\text{lysate}} \right)}} \times 100$$

### Measurement of histamine release

RBL-2H3 cells (5 × 10^4^ cells/well) in a 24-well plate were cultured for 24 h, and washed with MEM without FBS. Cells were incubated with different concentrations of sample solutions for 30 min at 37 °C in an atmosphere 5% CO_2_. Then, the supernatants were collected and centrifuged at 845*g* for 10 min. Histamine content in the supernatant was determined using ELISA histamine kits according to the manufacturer’s instructions.

Mice were randomly divided into eight groups (n = 8), including vehicle control, C48/80 (2.5 mg/kg), Rd groups (10, 20 and 40 mg/kg) and 20(*S*)-Rg3 groups (5, 10 and 20 mg/kg). All test compounds were dissolved in saline containing 20% propylene glycol and continuously injected via the tail vein. Blood was collected at 10 min, and serum was obtained through centrifugation (825*g*, 10 min, room temperature). Histamine content in the supernatant was determined using ELISA histamine kits according to the manufacturer’s instructions.

### Fluorescence microscopy

According to the instructions provided with the Annexin V-FITC detection kit, RBL-2H3 cells were treated with different sample solutions for 30 min and washed twice with cold PBS. Then binding buffer containing Annexin V-FITC (5 μl) was added. After 5 min in the dark, cells were examined and photographed at 630× magnification on an laser scanning confocal microscope (Zeiss LSM700, Jena, Germany).

### Measurement of intracellular Ca^2+^ concentration ([Ca^2+^]_i_)

[Ca^2+^]_i_ was measured using the fluorescence indicator calcium 5/AM. MPMCs or LAD2 cells were plated onto a 96-well plate coated with 50 μg/ml PDL and balanced at 37 °C in an atmosphere of 5% CO_2_. After 6 h, cells were loaded with calcium-5 with 0.02% Pluronic F-127 for 45 min at 37 °C in an atmosphere of 5% CO_2_, and washed five times with K25-Locke’s buffer (4 mM NaHCO_3_, 10 mM HEPES, 5 mM glucose, 2.3 mM CaCl_2_, 1 mM MgCl_2_ and 134 mM NaCl/25 mM KCl, pH 7.4 at 37 °C) [17], to leave a final volume of 150 μl in each well. Then, the plate was transferred to the plate chamber of FLIPR (Molecular Devices, Sunnyvale, CA, USA). Cells were excited at 470–495 nm, and emission detected at 515–575 nm. Fluorescence readings were taken every 1 for 120 s to establish the baseline. Then, 50 μl of different concentrations of sample solutions (4×) were added to different wells from the compound plate at 26 μl/s, yielding a final volume of 200 μl/well. Fluorescence readings were taken for 20 min.

### Statistical analyses

All analyses were undertaken using GraphPad Prism v5.01 (GraphPad Software, La Jolla, CA, USA). Data are the mean ± SEM. One-way analysis of variance followed by Dunnett’s test was used for multiple comparisons. *p* < 0.05 was considered significant. Each experiment was repeated at least thrice.

## Results

### RGE induced release of β-hexosaminidase from RBL-2H3 cells

**Fig. 1:**
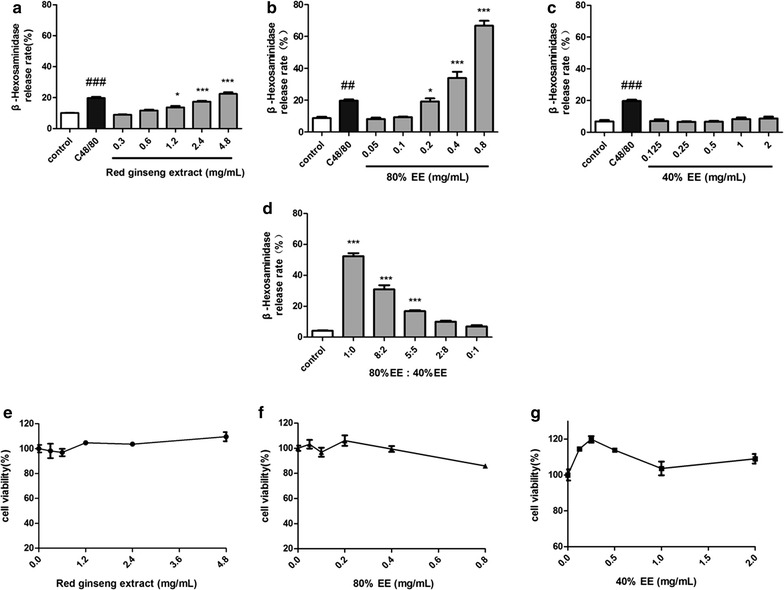
Effect of different extracts on the β-hexosaminidase release and viability of RBL-2H3 cells. RBL-2H3 cells were treated with different concentrations of extracts for 30 min and β-hexosaminidase release and cell viability were determined. **a**–**d** Effect of different extracts on the β-hexosaminidase release of RBL-2H3 cells. **e**–**g** Effect of different extracts on viability of RBL-2H3 cells by MTT assay. *p < 0.05, ***p < 0.001, compared with control group. ^##^ *p* < 0.01, ^###^ *p* < 0.001, positive control C48/80 compared with control group

Degranulation was monitored by β-hexosaminidase release. RGE (Fig. 1a) could induce β-hexosaminidase release in RBL-2H3 cells directly at >1.2 mg/ml. 80% EE (Fig. 1b) could induce significant release (*p* < 0.05) of β-hexosaminidase at 0.2 mg/ml in a concentration-dependent manner, whereas 40% EE (Fig. 1c) could not. Change in β-hexosaminidase release by combination of different ratios of 80% EE and 40% EE (Fig. 1d) was dependent upon the proportion of 80% EE. These findings suggested that RGE might possess allergenicity and that allergens might be present in 80% EE.

### Release of β-hexosaminidase from RBL-2H3 cells was due to allergenicity rather than cytotoxicity

To investigate if β-hexosaminidase release was associated with cytotoxicity, cell viability was determined by the MTT assay simultaneously. After incubation with different concentrations of RGE (Fig. 1e), 80% EE (Fig. 1f) and 40% EE (Fig. 1g), there was virtually no effect on cell viability, suggesting that cell degranulation was induced by allergenicity rather than cytotoxicity.

### HPLC–DAD-Q-TOF–MS/MS analyses of the RBL-2H3-binding components of RGE

**Fig. 2:**
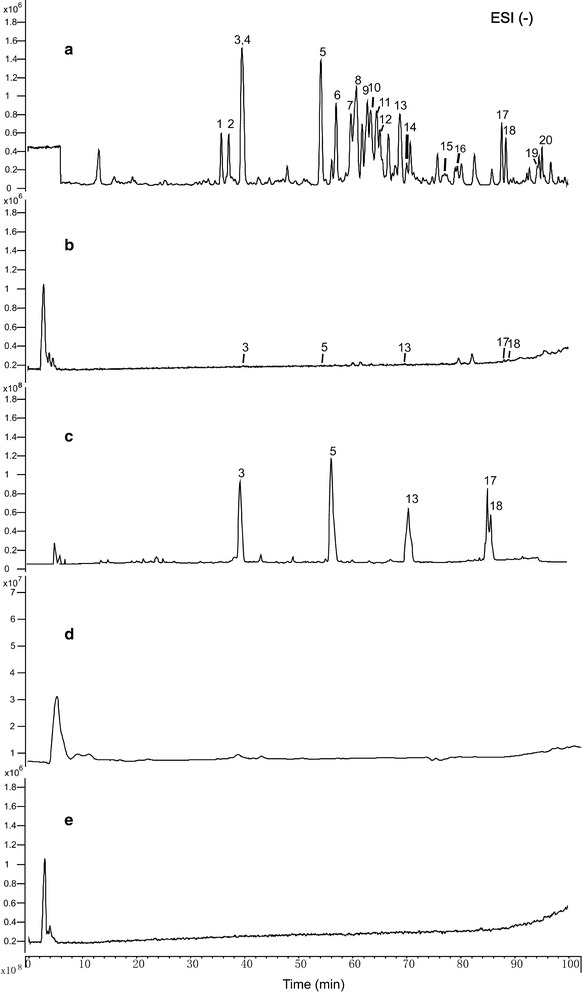
Total ion chromatograms of different samples in negative ion mode. Total ion chromatograms of different samples were analyzed by HPLC–DAD-Q-TOF–MS/MS. **a** RGE, **b** cell disruption, **c** reference substances, **d** control sample, **e** final-wash eluate. *3* Rg1, *5* Rf, *13* Rd, *17* 20(*S)*-Rg3, *18* 20(*R*)-Rg3

**Fig. 3:**
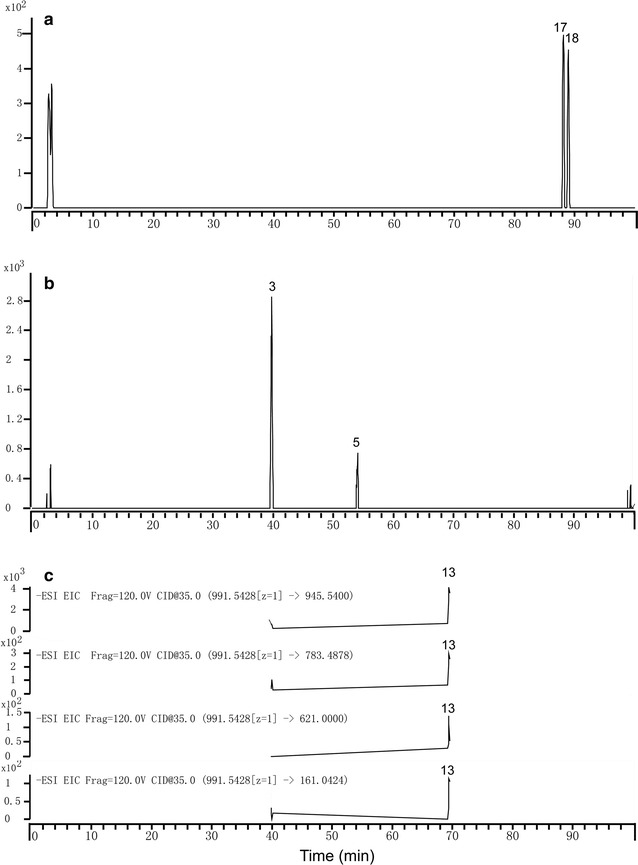
Extract ion chromatograms of RBL-2H3-binding components in negative ion mode. Five components were screened by extract ion chromatograms and comparing the mass spectra with reference substances solution: **a** 20(*S*)-Rg3 and 20(*R*)-Rg3, **b** Rg1 and Rf, **c** Rd

RBL-2H3 cell extraction was used to screen the components binding to RBL-2H3 cells. Twenty major ginsenosides were identified from RGE (Fig. 2a), nine of which [Rg1, Rb1, Re, Rf, Rg2, Rd, 20(*S*)-Rg3, 20(*R*)-Rg3, Ro] were confirmed by comparing the mass spectra and retention times with those of reference compounds, whereas the others were tentatively those of known ginsenosides. Details of identified ginsenosides are summarized in Table 1. RBL-2H3-binding components were screened and identified by extracted ion chromatograms (EIC) mode. Five components (peaks 3, 5, 13, 17, 18) were screened (Fig. 2b) by EIC mode and comparing the mass spectra with reference substances (Fig. 2c): ginsenoside Rg1, Rf, 20(*S*)-Rg3, 20(*R*)-Rg3 and Rd. No components were detected in control sample (Fig. 2d) and final-wash eluate (Fig. 2e). 20(*S*)-Rg3 and 20(*R*)-Rg3 (Fig. 3a) were extracted simultaneously at *m/z* 783.4748 ([M−H]^−^). Rg1 and Rf (Fig. 3b) were extracted simultaneously at *m/z* 845.4896 ([M+HCOO]^−^). Rd (Fig. 3c) was extracted at *m/z* 945.5400, 783.4878, 621.0000 and 161.0424 based on *m/z* 991.5428, respectively.

**Table 1:** Components identified from RGE

No.	t_R_	ID	Molecular formula	[M+HCOO]^−^ or [M−H]^−^	MS/MS fragmentations in the negative mode with the energy 35 V CID
1	35.826	Noto R3	C_48_H_82_O_19_	1007.5483 [M+HCOO]^−^ 961.5432 [M−H]^−^	1007.5483 [M+HCOO]^−^; 961.5432 [M−H]^−^; 799.4912 [M−H–Glc]^−^; 637.4346 [M−H–GlcGlc]^−^; 475.3805 [M−H–GlcGlcGlc]^−^; 323.0892 [GlcGlc–H]^−^
2	37.206	Noto R1	C_47_H_80_O_18_	977.5363 [M+HCOO]^−^ 931.5303 [M−H]^−^	977.5363 [M+HCOO]^−^; 931.5303 [M−H]^−^; 799.4886 [M−H–Xyl]^−^; 637.4339 [M−H–Xyl–Glc]^−^; 475.3798 [M−H–Xyl–GlcGlc]^−^; 293.0900 [XylGlc–H_2_O–H]^−^; 179.0552 [Glc–H]^−^
3	39.586	Rg1	C_42_H_72_O_14_	845.5033 [M+HCOO]^−^ 799.4892 [M−H]^−^	845.5033 [M+HCOO]^−^; 799.4892 [M−H]^−^; 637.4386 [M−H–Glc]^−^; 475.3836 [M−H–GlcGlc]^−^; B_1α_/B_1β_ 161.0445
4	40.000	Re	C_48_H_82_O_18_	991.5560 [M+HCOO]^−^ 945.5464 [M−H]^−^	991.5560 [M+HCOO]^−^; 945.5464 [M−H]^−^; 783.4931 [M−H–Glc]^−^; 637.4366 [M−H–Glc–Rha]^−^; 475.3818 [M−H–GlcGlc–Rha]^−^
5	54.282	Rf	C_42_H_72_O_14_	845.5028 [M+HCOO]^−^ 799.4923 [M−H]^−^	845.5028 [M+HCOO]^−^; 799.4923 [M−H]^−^; 637.4379 [M−H–Glc]^−^; 475.3831 [M−H–GlcGlc]^−^; 391.2842 [M−H–GlcGlc–C_6_H_12_]^−^; ^1,3^A_2β_ 221.0648; ^2,5^A_1β_ 101.0244
6	57.123	Noto R2	C_41_H_70_O_13_	815.4884 [M+HCOO]^−^ 769.4815 [M−H]^−^	815.4884 [M+HCOO]^−^; 769.4815 [M−H]^−^; 637.4377 [M−H–Xyl]^−^; 475.3834 [M−H–Xyl–Glc]^−^; 391.2905 [M−H–Xyl–Glc–C_6_H_12_]^−^
7	59.815	Rg2	C_42_H_72_O_13_	829.5027 [M+HCOO]^−^ 783.4934 [M−H]^−^	829.5027 [M+HCOO]^−^; 783.4934 [M−H]^−^; 637.3811 [M−H–Rha]^−^; 475.3811 [M−H–Rha–Glc]^−^; 391.2858 [M−H–Rha–Glc–C_6_H_12_]^−^
8	60.838	Rb1	C_54_H_92_O_23_	1107.5955 [M−H]^−^	1107.5955 [M−H]^−^; 945.5485 [M−H–Glc]^−^; 783.4839 [M−H–GlcGlc]^−^
9	62.804	Rc	C_53_H_90_O_22_	1077.5876 [M−H]^−^	1077.5876 [M−H]^−^; 945.5405 [M−H–Ara(f)]^−^; 783.4902 [M−H–Ara–Glc]^−^; 621.4365 [M−H–Ara–Glc–Glc]^−^; 459.4026 [M−H–Ara–GlcGlcGlc]^−^
10	63.459	Ro	C_48_H_76_O_19_	955.4959 [M−H]^−^	955.4959 [M−H]^−^; 793.4324 [M−H–Glc]^−^; ^0,2^A_1β_ 119.0318
11	64.666	Rb2	C_53_H_90_O_22_	1077.5868 [M−H]^−^	1077.5868 [M−H]^−^; ^2,5^A_1β_ 101.7272; ^0,4^A_2α_ 191.0518
12	65.218	Rb3	C_53_H_90_O_22_	1077.5876 [M−H]^−^	1077.5876 [M−H]^−^; 945.537 [M−H–Xyl]^−^; 783.4955 [M−H–Xyl–Glc; 621.4405 [M−H–Xyl–GlcGlc]^−^; B_2α_ 293.0941 [XylGlc–H]^−^; C_1α_ 149.0445 [Xyl–H]^−^
13	68.840	Rd	C_48_H_82_O_18_	991.5492 [M+HCOO]^−^ 945.4906 [M−H]^−^	991.5492 [M+HCOO]^−^; 945.4906 [M−H]^−^; 783.4501 [M−H–Glc]^−^; 621.4348 [M−H–GlcGlc]^−^; B_1α_/B_1β_ 161.0439 [Glc–H]^−^; ^2,5^A_1α_/^2,5^A_1β_ 101.0234
14	70.129	mRd	C_51_H_84_O_21_	1031.5434 [M−H]^−^	1031.5434 [M−H]^−^; 945.5438 [M−H–mal]^−^; 83.4890 [M−H–mal–Glc]^−^; 621.4369 [M–mal–H–GlcGlc]^−^; 459.3829 [M–mal–H–GlcGlcGlc]^−^; B_1α_/B_1β_ 161.0433 [Glc–H_2_O–H]^−^
15	77.568	Rg4/Rg6	C_42_H_70_O_12_	811.4891 [M+HCOO]^−^ 765.4832 [M−H]^−^	811.4891 [M+HCOO]^−^; 765.4832 [M−H]^−^; 457.3645 [M−H–Rha–Glc]^−^; ^1,3^A_2β_ 205.0715; ^0,2^A_1β_ 101.0231
16	79.547	Rg4/Rg6	C_42_H_70_O_12_	811.4891 [M+HCOO]^−^ 765.4832 [M−H]^−^	811.4891 [M+HCOO]^−^; 765.4832 [M−H]^−^; 457.3645 [M−H–Rha–Glc]^−^; ^1,3^A_2β_ 205.0715; ^0,2^A_1β_ 101.0231
17	87.753	20(*S*)-Rg3	C_42_H_72_O_13_	829.5002 [M+HCOO]^−^ 783.4932 [M−H]^−^	829.5002 [M+HCOO]^−^; 783.4932 [M−H]^−^; 621.4402 [M−H–Glc]^−^; 459.3860 [M−H–Glc–Glc]^−^; ^1,3^A_2β_ 221.0662; B_1β_ 161.0445 [Glc–H]^−^
18	88.547	20(*R*)-Rg3	C_42_H_72_O_13_	829.4991 [M+HCOO]^−^ 783.4931 [M−H]^−^	829.4991 [M+HCOO]^−^; 783.4931 [M−H]^−^; 621.4377 [M−H–Glc]^−^; 459.3846 [M−H–GlcGlc]^−^; ^1,3^A_2β_ 221.0635; B_1β_ 161.0427 [Glc–H_2_O–H]^−^
19	94.527	Rg5/Rk1	C_42_H_70_O_12_	765.4816 [M−H]^−^	765.4816 [M−H]^−^; 603.4279 [M−H–Glc]^−^; 441.3775 [M−H–GlcGlc]^−^; ^1,3^A_2β_ 221.0643; B_1β_ 161.0428 [Glc–H_2_O–H]^−^
20	95.286	Rg5/Rk1	C_42_H_70_O_12_	765.4816 [M−H]−	765.4816 [M−H]^−^; 603.4279 [M−H–Glc]^−^; 441.3775 [M−H–GlcGlc]^−^; ^1,3^A_2β_ 221.0643; B_1β_ 161.0428 [Glc–H2O–H]^−^

### Ginsenoside Rd and 20(*S*)-Rg3 induced β-hexosaminidase release from RBL-2H3 cells

**Fig. 4:**
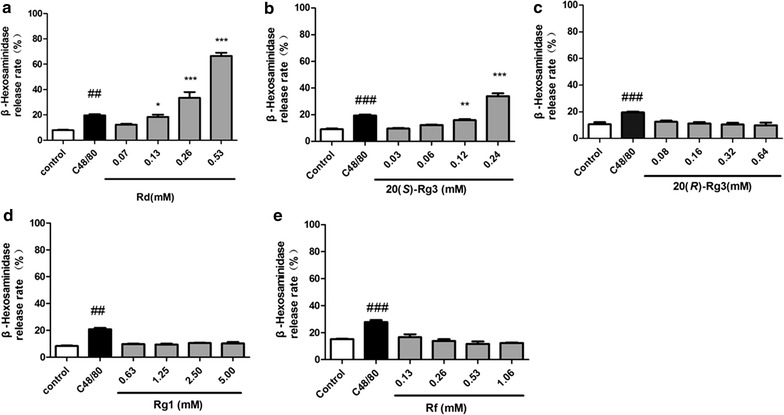
Effect of binding components on β-hexosaminidase release from RBL-2H3 cells. RBL-2H3 cells were treated with different concentrations of binding components for 30 min and β-hexosaminidase release was examined. **a** Rd, **b** 20(*S*)-Rg3, **c** 20(*R*)-Rg3, **d** Rg1, **e** Rf. *p < 0.05, **p < 0.01, ***p < 0.001, compared with control group. ^#^ *p* < 0.05, ^##^ *p* < 0.01, ^###^ *p* < 0.001, positive control C48/80 compared with control group

Due to the possibility of non-selective binding, the effect of screening of each component mentioned above on β-hexosaminidase release from RBL-2H3 cells was determined to verify allergenicity preliminarily. Rd (Fig. 4a) and 20(*S*)-Rg3 (Fig. 4b) could induce direct degranulation of MCs in a concentration-dependent manner, whereas 20(*R*)-Rg3 (Fig. 4c), Rg1 (Fig. 4d), and Rf (Fig. 4e) had no significant effect on MCs (*p* > 0.05). These results suggested that ginsenoside 20(*S)*-Rg3 and Rd might possess allergenicity to some extent.

### Ginsenoside Rd and 20(*S*)-Rg3 induced histamine release from RBL-2H3 cells

**Fig. 5:**
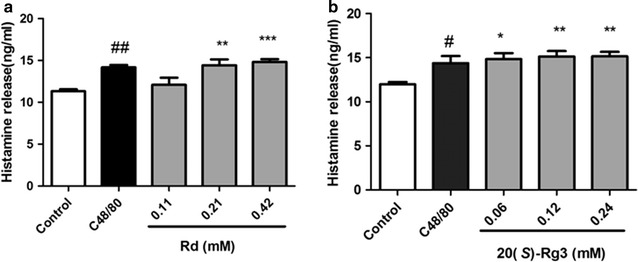
Effect of Rd and 20(*S*)-Rg3 on histamine release from RBL-2H3 cells. RBL-2H3 cells were treated with different concentrations of Rd and 20(*S*)-Rg3 for 30 min and histamine release was determined by ELISA histamine kits. **a** Rd, **b** 20(*S*)-Rg3. *p < 0.05, **p < 0.01, ***p < 0.001, compared with control group. ^#^ *p* < 0.05, ^##^ *p* < 0.01, positive control C48/80 compared with control group

Histamine is also a classic mediator released concomitantly with β-hexosaminidase if MCs are activated. Histamine release in supernatants of RBL-2H3 cells was determined using an ELISA kit. Ginsenoside Rd (Fig. 5a) and 20(*S*)-Rg3 (Fig. 5b) could also induce significant release of histamine, which was consistent with that seen with β-hexosaminidase.

### Ginsenoside Rd and 20(*S*)-Rg3 induced phosphatidylserine translocation in RBL-2H3 cells

**Fig. 6:**
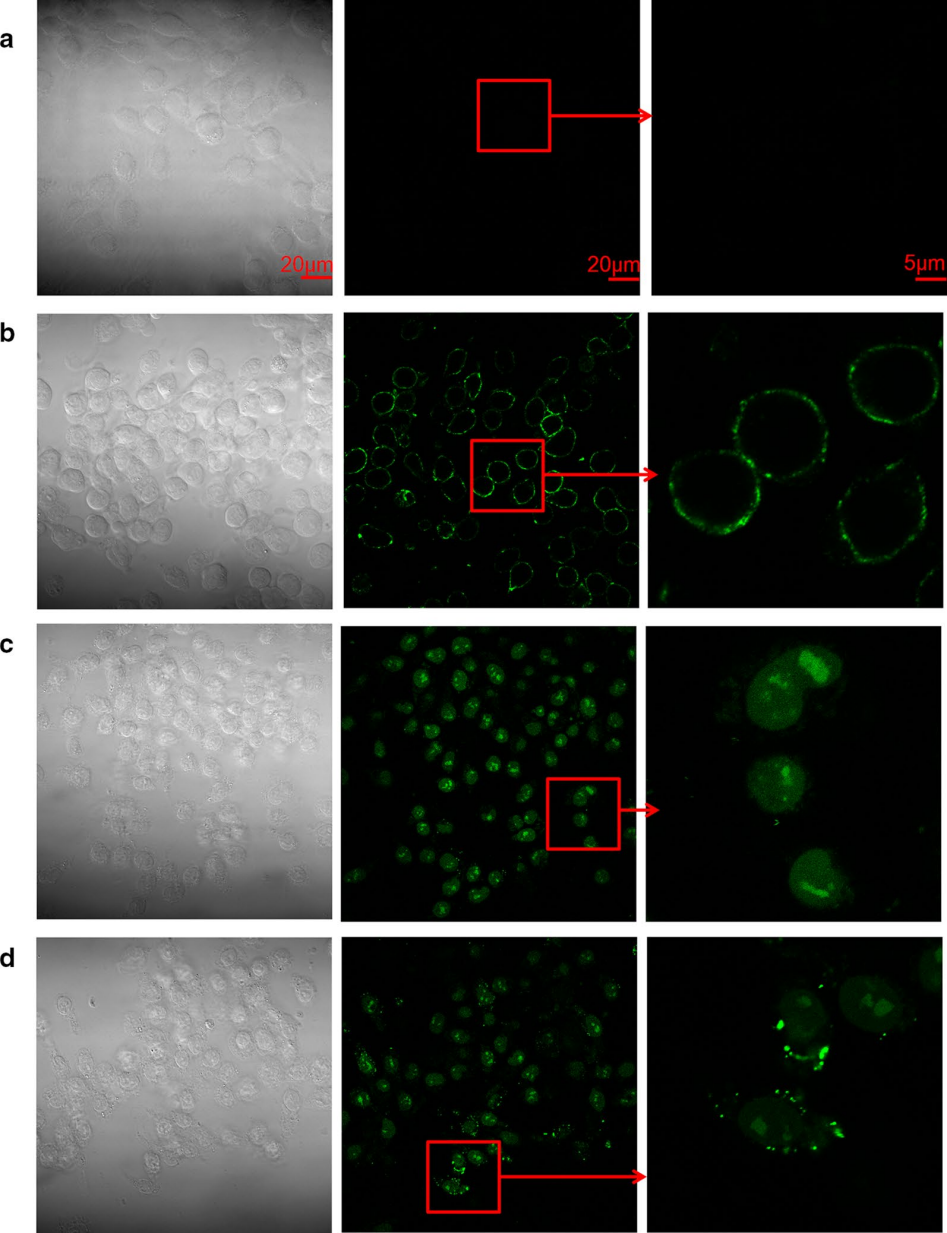
Effect of potential allergens on phosphatidylserine translocation in RBL-2H3 cells. RBL-2H3 cells were treated with different concentrations of test compounds for 30 min, and phosphatidylserine translocation was determined by an Annexin V-FITC fluos staining kit. Cells were photographed at ×630 magnification on an laser scanning confocal microscope. **a** vehicle, **b** C48/80 (30 μg/ml), **c** Rd (0.43 mM), **d** 20(*S*)-Rg3 (0.25 mM)

It has been reported that the membrane phospholipid phosphatidylserine translocates from the inner to outer leaflets of cytomembranes during MC degranulation [18, 19]. Annexin V-FITC can bind to cells with exposed phosphatidylserine, causing the cells to appear green. Compared with the vehicle group (Fig. 6a) and C48/80 group (Fig. 6b), ginsenoside Rd (Fig. 6c) and 20(*S*)-Rg3 (Fig. 6d) could induce phosphatedylserine translocation in RBL-2H3 cells.

### Ginsenoside Rd and 20(*S*)-Rg3 induced β-hexosaminidase release from MPMCs and LAD2 cells

**Fig. 7:**
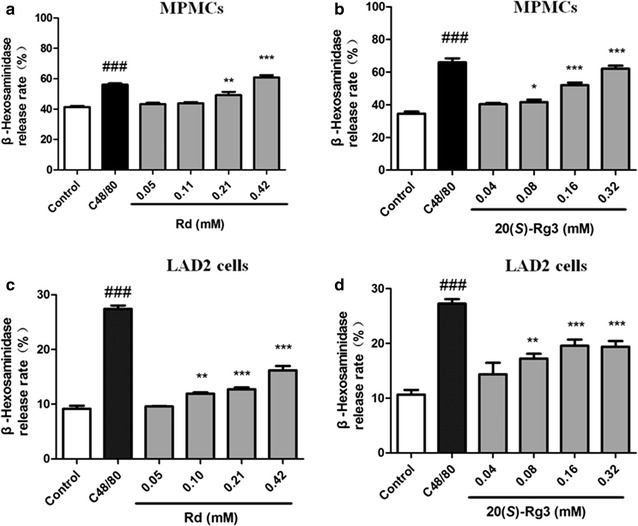
Effect of Rd and 20(*S*)-Rg3 on β-hexosaminidase release from MPMCs (**a**, **b**) and LAD2 cells (**c**, **d**). MPMCs were treated with different concentrations of Rd and 20(*S*)-Rg3 for 30 min and β-hexosaminidase release was determined. **a** Effect of Rd on MPMCs. **b** Effect of 20(*S*)-Rg3 on MPMCs. **c** Effect of Rd on LAD2 cells. **d** Effect of 20(*S*)-Rg3 on LAD2 cells. *p < 0.05, **p < 0.01, ***p < 0.001, compared with control group. ^###^ *p* < 0.01, positive control C48/80 compared with control group

To verify further the reliability of the results obtained in RBL-2H3 cells, the same indicators in MPMCs and LAD2 cells were determined. Release of β-hexosaminidase induced by Rd and 20(*S*)-Rg3 in MPMCs (Fig. 7a, b) and LAD2 cells (Fig. 7c, d) was consistent with that observed in RBL-2H3 cells.

### Ginsenoside Rd and 20(*S*)-Rg3 induced an increase of [Ca^2+^]_i_ of MPMCs and LAD2 cells

**Fig. 8:**
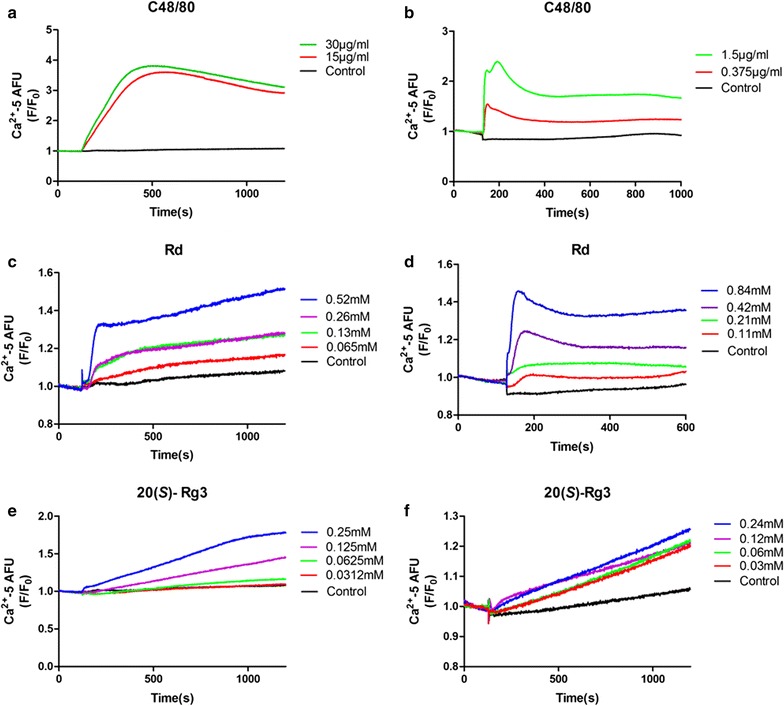
Effect of potential allergens on [Ca^2+^]_i_ of MPMCs (**a**, **c**, **e**) and LAD2 cells (**b**, **d**, **f**). Cells were loaded with calcium-5 with 0.02% Pluronic F-127, and fluorescence readings were taken every 1 s for 120 s to establish the baseline. Then, different concentrations of sample solutions were added to different wells from the compound plate, and fluorescence readings were taken for 20 min

Increase in cytosolic calcium is essential both for degranulation and the release of de novo synthesized mediators in mast cells [20]. Change in [Ca^2+^]_i_ is an indication of the degree of mast cell degranulation. So we investigated if Rd and 20(*S*)-Rg3 could induce Ca^2+^ mobilization in MPMCs and LAD2 cells. C48/80 (Fig. 8a, b), ginsenoside Rd (Fig. 8c, d) and 20(*S*)-Rg3 (Fig. 8e, f) could induce responses in MPMCs and LAD2 cells. The degree of response was dependent upon concentration, which suggested that the mechanism of MC activation induced by ginsenoside Rd and 20(*S*)-Rg3 might be related to an increase in [Ca^2+^]_i_.

### Ginsenoside Rd and 20(*S*)-Rg3 induced an increase of histamine in mice serum

**Fig. 9:**
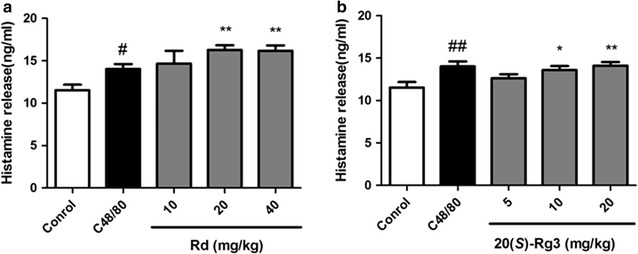
Effect of Rd and 20(*S*)-Rg3 on serum histamine level in mice at 10 min post-injection. After 10 min of intravenous injection, the histamine levels in mice serum of the Rd groups (**a**) and 20(*S*)-Rg3 groups (**b**) were determined by ELISA histamine kits. *p < 0.05, **p < 0.01, compared with control group. ^#^ *p* < 0.01, ^##^ *p* < 0.01, positive control C48/80 compared with control group

Our results indicated that just after 10 min of intravenous injection, the histamine levels in serum of the Rd groups (Fig. 9a) and 20(*S*)-Rg3 groups (Fig. 9b) were elevated dose-dependently compared with that of the vehicle control group, and similar effect was observed in the positive control C48/80 group. These results showed that Rd and 20(*S*)-Rg3 exhibited potential anphylactoid activity in vivo.

## Discussion

Recently, reports about ARs induced by red ginseng have been increasing, but opinions on the source of allergens have been ambiguous. Solubilizers, such as Tween-80, have been reported to induce ARs in vitro or in vivo [21]. It has also been reported that red ginseng itself might induce MC degranulation [9]. However, a systematic study on the allergenicity and allergens related to ginsenosides in red ginseng is lacking. In the current study, we found that RGE could induce β-hexosaminidase release from RBL-2H3 cells, and that allergens were present mainly in 80% EE. Five components were screened by RBL-2H3 cell extraction and identified by EIC mode: ginsenoside Rd, 20(*S*)-Rg3, 20(*R*)-Rg3, Rg1 and Rf. We demonstrated that Rd and 20(*S*)-Rg3 were potential pseudo-allergenic ingredients in red ginseng by further verification in MPMCs, LAD2 cells (the only human analogue [22]) and mice. This is the first systematic evaluation on allergenicity and screening of potential pseudo-allergic ingredients of RGE, and ginsenoside 20(*S*)-Rg3 is reported to induce MC activation for the first time in our study. 20(*S*)-Rg3 is one of characteristic components in red ginseng, so the results further demonstrate that red ginseng is more likely to induce ARs than other processed products. The results about Rd allergenicity are consistent with the study by Lu et al. [23]. They demonstrated that Rd was the direct inductor of RBL-2H3 degranulation. Therefore, our study also confirms the reliability of RBL-2H3 cell extraction as a method of preliminarily screening potential allergens.

ARs, which usually occur upon application of drugs or functional foods, are a serious public-health concern [24]. They are not dependent on specific IgE and do not necessarily require previous exposure to an inciting substance. However, they are always dose- and concentration-dependent. Allergens can stimulate MCs or basophils directly to release biologically active substances via physical or chemical mechanisms that are not based on the immune system, though the true mechanism of action still remains unclear. β-Hexosaminidase is a potent inflammatory mediator stored in MCs and basophils and is often released in parallel with histamine from activated MCs and basophils. β-Hexosaminidase release is slow and the process persists for a longer time than that observed for histamine, so it is often used as a substitute for direct quantification of histamine during cell degranulation processes [25]. The membrane phospholipid phosphatidylserine translocates from the inner to outer leaflets of the cytomembranes if mast cell degranulation occurs [18]. Also, elevation of [Ca^2+^]_i_ occurs during degranulation, which is also involved in activation of tumor necrosis factor α, IL-1β and IL-6 [17, 26].

In studies on the safety of TCMs or various preparations, most studies have focused on the properties of pharmaceutical adjuvants or food additives, whereas that the safety of the bioactive components themselves has attracted increasing attention in recent years. Several bioactive components have been reported to possess potential safety risks, such as bacalin [27], chlorogenic acid, cryptochlorogenic acid [28] and neoandrographolide [29], have also been reported to cause ARs by inducing MC degranulation directly in vitro or in vivo. Therefore, safety evaluation and improvement of quality requires more attention. Additionally, more effective methods should be established to screen target components in the complex system of TCMs quickly.

Ginsenoside Rd and 20(*S*)-Rg3 are bioactive components and reported to possess anti-inflammation [30, 31] and anti-cancer activities [32], respectively. However, Rg3 has been reported to induce irreversible impairment of agonist-induced vascular contraction through blockade of Ca^2+^ mobilization via the L-type Ca^2+^ channel [33]. Rd may induce ARs by disturbing lipid metabolism [23]. [Ca^2+^]_i_ in MCs and basophils increases through signaling after activation, and then results in degranulation [34]. We found that Rd and 20(*S*)-Rg3 could induce an increase in [Ca^2+^]_i_ as could C48/80 (a classical MC activator and a canonical basic secretagogue). However, the mode of change in [Ca^2+^]_i_ seemed to be different, which suggested that upstream mechanisms of MC cell activation might be not identical. Rd and 20(*S*)-Rg3 are protopanaxodiol ginsenosides, and 20(*S*)-Rg3 can be transformed from Rd by a deglucosylation pathway in vivo [35]. Our results indicated that ginsenosides involved in the metabolic pathway of Rd might elicit ARs or show allergenicity to different extent.

Five components were screened in RBL-2H3 cell extraction, but only Rd and 20(*S*)-Rg3 could activate MCs and induce the significant release of β-hexosaminidase in a concentration-dependent manner. This phenomenon could result from non-selective absorption of cells. Conversely, RBL-2H3 cells can be used as a cell model of allergic reactions or anti-allergic reactions [36], anti-allergic bioactive components may have been absorbed by cells. Components were absorbed in RBL-2H3 cells but showed no potential for ARs were different to Rd and 20(*S*)-Rg3 in structure, so their anti-allergic bioactivities could be studied further.

## Conclusions

In conclusion, we supply a method, RBL-2H3 cell extraction, to screen potential allergens in complex components, and ginsenoside Rd and 20(*S*)-Rg3 are identified as potential allergens in red ginseng extract that may induce anaphylactoid reaction. Our study could guide optimization of methods associated with Rd/20(*S*)-Rg3-containing preparations and establishment of quality standards for safe application of TCMs.

## Abbreviations

ARs: anaphylactoid reactions
EIC: extracted ion chromatograms
IgE: immunoglobulin E
MCs: mast cells
MEM: modified Eagle’s medium
MPMC: mouse peritoneal mast cell
MTT: 3-(4,5-Dimethylthiazol-2-yl)-2,5-diphenylte-trazolium bromide
PBS: phosphate-buffered saline
PDL: poly-d-lysine hydrobromide
Q-TOF MS/MS: quadrupole time-of-flight tandem mass spectrometry
RGE: red ginseng extract
TCM: Traditional Chinese Medicine
TCMI: Traditional Chinese Medicine Injection
80% EE: 80% ethanol extract
40% EE: 40% ethanol extract
